# Concurrent high-intensity interval plus resistance training improves vascular health in breast cancer survivors with high chemotherapy exposure

**DOI:** 10.1186/s40659-026-00677-y

**Published:** 2026-02-23

**Authors:** Cristian Álvarez, Carolina Fuentes, Cristóbal Durán-Marín, Pedro Delgado-Floody, Gabriel Rojas-Rojas, Manuel Gomez, Alvaro N. Gurovich, David C. Andrade

**Affiliations:** 1 Exercise and Rehabilitation Sciences Institute, School of Physical Therapy, Faculty of Rehabilitation Sciences, Universidad Andres Bello, Santiago, 7591538 Chile; 2 School of Kinesiology, Universidad Andres Bello, Santiago, Chile; 3 Exercise and Rehabilitation Sciences Institute, Doctorate in Rehabilitation Science Program, Faculty of Rehabilitation Sciences, Universidad Andres Bello, Santiago, Chile; 4 Department of Physical Education, Sport and Recreation, Universidad de La Frontera, Temuco, Chile; 5 Clinical Applied Physiology (CAPh) Laboratory, Departmente of Physical Therapy and Movement Sciences, College of Health Sciences, The University of Texas at El Paso, El Paso, TX 79968 USA; 6 Exercise Applied Physiology Laboratory, Centro de Investigación en Fisiología y Medicina de Altura (FIMEDALT), Departamento Biomédico, Facultad de Ciencias de la Salud, Universidad de Antofagasta, Av. Universidad de Antofagasta #02800, Antofagasta, Chile

**Keywords:** Cancer, Breast cancer, Pulse wave velocity, Flow–mediated dilation, Carotid intima–media thickness, Concurrent training

## Abstract

**Background:**

The aim was to determine the effects of 8 weeks of concurrent training of abbreviated high-intensity interval-training and low–load resistance training (CT_HIIT+RT_) on functional and structural vascular outcomes of breast cancer survivors with a history of high or low exposure to chemotherapy sessions.

**Methods:**

Breast cancer survivor women (*n* = 21, 58.7 ± 8.7 years) were divided into high– (HV_chemo_, *n* = 11) or low–volume chemotherapy groups (LV_chemo_, *n* = 10). Pulse wave velocity (PWV) and carotid intima–media maximum (cIMT_max_) were the primary outcomes, while flow-mediated dilation (FMD), carotid intima–media average (cIMT_av_), baseline brachial artery diameter (D_base_), peak diameter (D_peak_), and one-repetition maximum tests for the biceps (1RM_bc_), shoulder (1RM_sp_), back (1RM_back_), and leg extension (1RM_Leg_) served as secondary outcomes.

**Results:**

PWV decreased in the LV_chemo_ group (∆˗1.64 m⋅s^− 1^, *p* < 0.001) but not in the HV_chemo_ group. cIMT_max_ was reduced only in the HV_chemo_ group (∆˗0.23 mm, *p* = 0.024). FMD was significantly increased in the HV_chemo_ (∆+5.06%, *p* = 0.023) but not in the LV_chemo_ group. HV_chemo_ and LV_chemo_ groups increased D_base_ (∆+0.38 mm, *p* = 0.005; and ∆+0.16 mm, *p* < 0.0001) and D_peak_ (∆+0.32 mm, *p* = 0.008; and ∆+0.12 mm, *p* < 0.0001). HV_chemo_ and LV_chemo_ groups increase 1RM_bc_ (∆+2.3 kg; and ∆+2.09 kg), 1RM_sp_ (∆+2.15 kg; and ∆+2.36 kg), 1RM_back_ (∆+4.4 kg; and ∆+4.58 kg), and 1RM_Leg_ (∆+6.15 kg; and ∆+5.27 kg), all *p* < 0.01.

**Conclusions:**

Eight weeks of CT_HIIT+RT_ in women breast cancer survivors who received higher chemotherapy volume reduces cIMT_max_, but increases FMD, D_base_, D_peak_, cardiorespiratory fitness, and muscle strength. Vascular improvements were partially observed in subjects with lower chemotherapy volumes.

**Supplementary Information:**

The online version contains supplementary material available at 10.1186/s40659-026-00677-y.

## Introduction

Cancer is the second leading cause of death worldwide after cardiovascular disease [1], and breast cancer accounts for ~ 12% of cases [2]. Accordingly, the World Health Organization launched the Global Breast Cancer Initiative (GBCI) to decrease breast cancer mortality by 2.5% annually over a 20–year period [3]. Worryingly, although in 2025 North America reports a higher incidence of breast cancer than Latin American countries (306,307 vs. 220,124 cases), mortality in Latin America is projected to remain higher by 2040 (117,967 vs. 90,765 deaths) [4]. Notably, the increased cancer risk has primarily been attributed to physical inactivity, unhealthy diet, obesity, and low income [5]. Therefore, GBCI’s mission is built on three central pillars: health promotion during pre-diagnostic and diagnostic intervals, and comprehensive breast cancer management during treatment [3].

Following diagnosis, treatments such as surgery (i.e., to remove the breast tumor), radiation (i.e., to decrease recurrence in breast tissues and surrounding areas), and pharmacological medications (i.e., to eliminate cancer cells), including hormonal therapy, targeted biological agents, and chemotherapy, are essential strategies during the acute survivorship stage. However, they may cause collateral effects [3]. Chemotherapy (chemo) has been associated with cardiotoxicity, chemotherapy-induced peripheral neuropathy [6], high blood pressure, arterial stiffness [7], and endothelial dysfunction, which compromises the ‘functional’ and ‘structural’ integrity of the endothelium and overall vascular health [8]. For example, it has been reported that pulse wave velocity (PWV), a marker for arterial stiffness and function, increases in patients after 6–12 months of chemo treatment by ~ 1 m·s^− 1^ [9]. Similarly, the *average* (cIMT_av_) and *maximum* intima-media thickness measurements (cIMT_max_) from the common carotid artery are important cardiovascular risk factors, with pharmacological and nutritional strategies reported to yield valuable reductions [10, 11]. Other studies suggest that after one month of completed chemo treatment, other vascular parameters, such as augmentation index, also worsen [9]. Furthermore, side effects of chemo treatment include fatigue, pain, anxiety, and decreased physical function. Additionally, other issues such as lymphedema, depressive symptoms, and bone health problems have also been reported [6].

Exercise training interventions have been shown to reduce cardiotoxicity and promote vascular remodeling, thereby mitigating the side effects of chemo [12]. Leading institutions such as the American College of Sports Medicine [6], the American Heart Association [13], and the American Diabetes Association [14] have recommended exercise training for adults undergoing chemo. Exercise has been demonstrated to reduce arterial stiffness. For example, resistance training (RT) enhances both function and the diameter (i.e., structure) of the brachial artery, and moderate-intensity continuous training (MICT) exerts similar effects on the femoral artery [15]. Furthermore, 12 weeks of RT reduced PWV by ~ 2.5 m·s^− 1^ [16], and moderate-to-high–intensity RT has been effective in preventing lymphedema in cancer survivors by reducing fluid accumulation [17]. Similarly, 12 weeks of MICT through walking or cycling led to significant reductions in arterial wall thickness in both popliteal and brachial arteries in adult men [18]. On the other hand, 8 weeks of high–intensity interval training (HIIT) showed no changes in the cIMT_av_ of breast cancer patients undergoing chemo treatment; however, these authors do not report the cIMT_max_ [19].

Other vascular exercise training effects include an increase in flow-mediated dilation (FMD), an endothelium-dependent nitric oxide bioavailability marker, induced after reactive hyperemia [20]. Sixteen weeks of concurrent training (CT), either CT_MICT+RT_ or CT_HIIT+RT_, showed that both modalities reduce fatigue and preserve physical function in breast cancer patients undergoing chemo [21]. Unfortunately, the authors did not include any vascular outcome. Therefore, besides the evidence supporting exercise training, there is limited evidence on the effectiveness of CT modalities, especially regarding CT_HIIT+RT_ on markers of vascular function and structure in breast cancer survivors. In particular, the effects of low–volume CT protocols, which combine short HIIT intervals (e.g., 60 s × ≤5 intervals) with RT in low–load conditions (e.g., 20–50% of 1RM), remain largely unexplored. Preliminary findings suggest that such protocols may lead to greater vascular improvements, including reduced arterial stiffness and enhanced vasodilatory response as measured by FMD [22, 23]. Nonetheless, the impact of CT_HIIT+RT_ remains poorly understood in cancer cohorts, especially regarding their role in reducing PWV or cIMTmax, which are associated with cardiotoxicity following chemotherapy. Therefore, this study aimed to evaluate the effects of 8 weeks of concurrent CT_HIIT+RT_ on the functional and structural vascular outcomes in breast cancer survivors with high or low exposure to chemo sessions. We hypothesized that CT_HIIT+RT_ can improve vascular function (as reflected by decreases in PWV or cIMT_max_).

## Results

The adherence reported was as follows: high-volume chemo (HV_chemo_) 15.2 sessions and low-volume chemo (LV_chemo_) 13.7 sessions. There was no statistical significance between groups.

### **Pre and post-training changes in anthropometric and body composition**

(Table 1) presents the anthropometric and body composition characteristics of the participants before and after the intervention, showing no significant differences between the HV_chemo_ and LV_chemo_ groups at baseline. Waist circumference significantly decreased in both HV_chemo_ (∆˗4.5 cm, *p* = 0.002, *d* 0.57) and LV_chemo_ group (∆˗6.6 cm, *p* = 0.002, *d* 0.71). However, the difference between groups remained nonsignificant (*p* = 0.221). The cardiorespiratory fitness was significantly increased in HV_chemo_ (∆+4.9 mL·kg·min^− 1^, *p* < 0.0001, *d* 0.81) and LV_chemo_ group (∆+3.9 mL·kg·min^− 1^, *p* < 0.0001, *d* 0.81). No differences were observed in body composition, body fat %, skeletal muscle mass, or fat-free mass within or between groups (Table 1).

**Table 1:** Anthropometric and body composition characteristics of participants before and after intervention

Outcomes	Time	HV_chemo_	LV_chemo_	Between-group Pvalue, Cohen d
*Anthropometric*				
Age (y)	Pre	58.1 ± 10.4	59.3 ± 7.0	*P* = 0.980
Height (m)	Pre	1.56 ± 0.03	1.56 ± 0.03	*P* = 0.766
Weight (kg)	Pre	70.8 ± 12.5	66.9 ± 11.2	*P* = 0.608, *d* 0.03
	Post	71.7 ± 12.4	66.9 ± 11.3	*P* = 0.495, *d* 0.05
	*P*value	*P* = 0.218, *d* 0.20	*P* = 0.805, *d* 0.00	
Body mass index (kg/m^2^)	Pre	28.8 ± 4.5	27.3 ± 5.3	*P* = 0.642, *d* 0.02
	Post	29.2 ± 4.4	27.3 ± 5.2	*P* = 0.515, *d* 0.04
	*P*value	*P* = 0.210, *d* 0.18	*P* = 0.822, *d* 0.02	
Waist circumference (cm)	Pre	102.8 ± 13.4	97.1 ± 11.5	*P* = 0.577, *d* 0.04
	Post	98.3 ± 11.4*	90.5 ± 12.6*	*P* = 0.221, *d* 0.18
	*P*value	***P*** **= 0.002**, ***d*** **0.57**	***P*** **= 0.002**, ***d*** **0.71**	
*Body composition*				
Body fat (%)	Pre	41.4 ± 4.3	37.8 ± 6.4	*P* = 0.219, *d* 0.16
	Post	41.8 ± 2.6	39.2 ± 5.0	*P* = 0.604, *d* 0.04
	*P*value	*P* = 0.117, *d* 0.31	*P* = 0.051, *d* 0.22	
Skeletal muscle mass (kg)	Pre	22.7 ± 3.4	22.5 ± 2.7	*P* = 0.953, *d* 0.00
	Post	23.4 ± 3.4	23.5 ± 1.9	*P* = 0.823, *d* 0.00
	*P*value	*P* = 0.613, *d* 0.29	*P* = 0.310, *d* 0.09	
Fat free mass (kg)	Pre	41.8 ± 5.6	41.3 ± 4.4	*P* = 0.949, *d* 0.00
	Post	42.9 ± 5.6	42.9 ± 3.1	*P* = 0.780, *d* 0.01
	*P*value	*P* = 0.070, *d* 0.31	*P* = 0.089, *d* 0.22	
*Cardiorespiratory fitness*				
Peak oxygen consumption (mL·kg·min^− 1^)	Pre	13.4 ± 2.2	15.7 ± 2.9	*P* = 0.127, *d* 2.3
	Post	18.3 ± 2.7	19.6 ± 3.3	*P* = 0.698, *d* 1.3
	*P*value	***P*** **< 0.0001**, ***d*** **0.81**	***P*** **< 0.0001**, ***d*** **0.63**	

Data are shown as mean and ± SD. Groups are described as (HV_chemo_) High-volume chemotherapy sessions, (LV_chemo_) Low-volume chemotherapy sessions. (*) Denotes significant within-group differences from pre-test at *P* ≤ 0.05. (*d*) Cohen d effect size.

### Pre–and post-training changes in vascular functional/structural (Main outcomes)

**Fig. 1:**
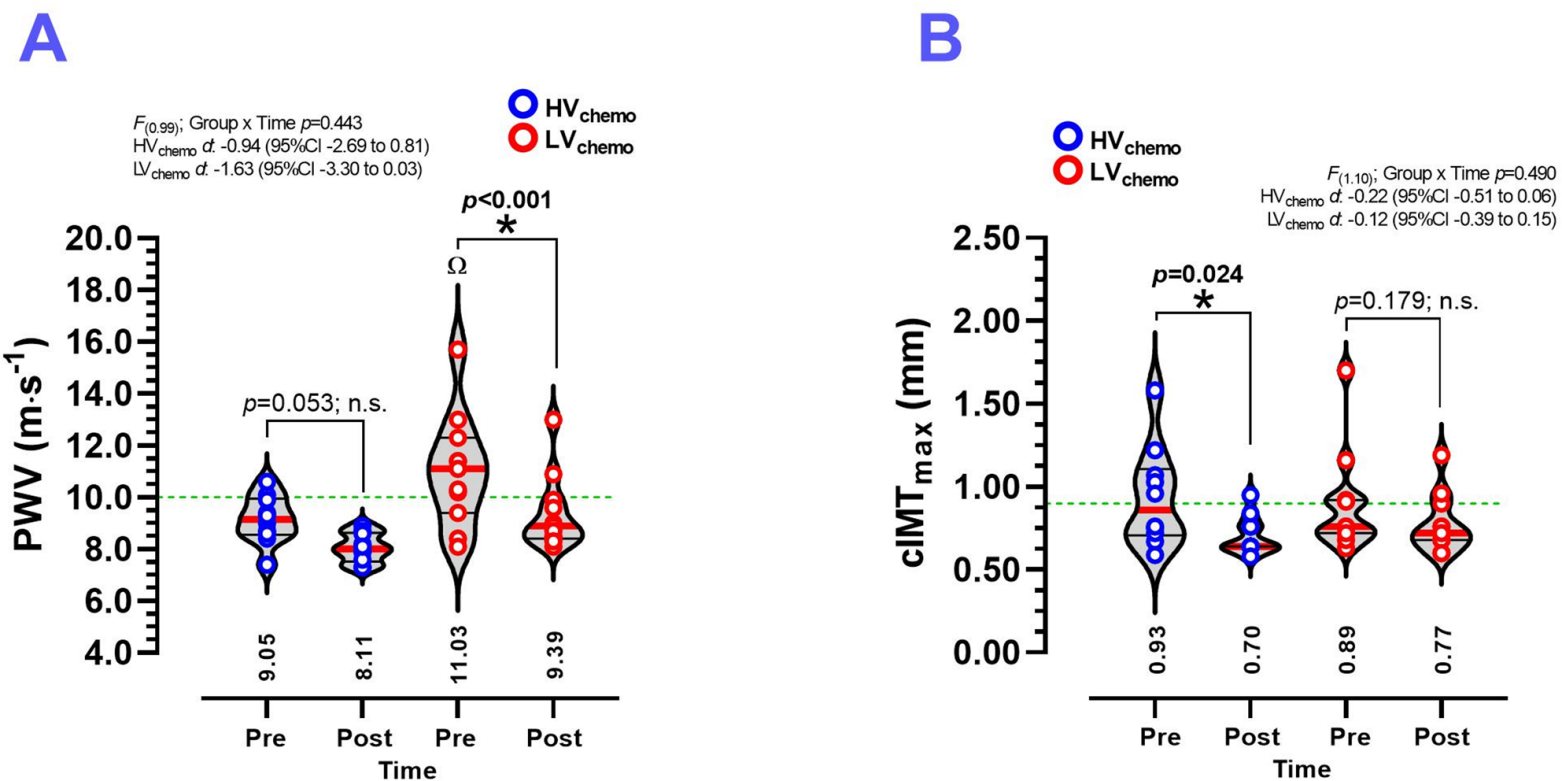
Pulse wave velocity (**A**) and carotid intima-media thickness *maximum* (**B**) measured before and after 8 weeks of concurrent training in women breast cancer survivors with history of high-volume and low-volume of chemotherapy treatment. **Groups are described as;** (HV_chemo_) High-volume chemotherapy group, (LV_chemo_) Low-volume chemotherapy group. **Outcomes are described as;** (PWV) Pulse wave velocity, and (cIMT_max_) Carotid intima-media thickness *maximum. *(n.s.) No significant modifications. (Ω) Denotes significant between-group baseline differences at *P* < 0.05. (*) Denotes significant differences between pre vs. post-test at *P* < 0.05. (*d*) Denotes Cohen d effects size with 95%CI. All bold values denote significant statistical changes/differences at *P* ≤ 0.05

At baseline, there were significant differences between the HV_chemo_ vs. the LV_chemo_ group (9.05 ± 1.00 vs. 11.03 ± 2.10 m⋅s-1, *p* < 0.05) (Fig. 1A). PWV was significantly decreased in LV_chemo_ (11.03 ± 2.10 to 9.39 ± 1.40 m⋅s-1, *p* < 0.001, d − 1.63) (Fig. 1A), while in the HV_chemo_ group did not show changes (Fig. 1A). The cIMT_max_ was significantly decreased only in the HV_chemo_ group (0.93 ± 0.30 to 0.70 ± 0.11 mm, *p* = 0.024, d − 0.22) (Fig. 1B), while cIMT_max_ in the HVchemo group did not show changes (Fig. 1B).

The interactions Group x Time, Group x blood pressure medication use (i.e., participants with blood pressure therapy, and those with no blood pressure therapy), and Group x Age (i.e., participants aged < 60 y, and those with age ≥ 60 years) have also been tested by mixed-model analyses (see Supplementary Fig. S1). In Model 1 (Supplementary Fig. S1A), no significant group × time interaction was observed. In Model 2 (Supplementary Fig. S1B), a significant group × Blood Pressure-therapy interaction was detected *F*_(4.12)_; *p* < 0.0001). Participants under blood pressure therapy, especially in the HV_chemo_ group, exhibited higher baseline PWV and experienced greater reductions after the intervention compared with those not receiving blood pressure therapy. In Model 3 (Supplementary Fig. S1C), the group × age interaction was significant *F*_(6.86)_; *p* = 0.029). Older participants (> 60 years), in the HV_chemo_ group, demonstrated elevated baseline PWV and larger reductions over time, whereas younger participants showed smaller changes. Panels D–F present the effects on cIMT_max_, where differences did not reach statistical significance (Supplementary Fig. S1D-F).

Individual changes in pulse wave velocity (PWV, m·s⁻¹) are available (see Supplementary Fig. S2). In the HV_chemo_ group (red bars), 8 out of 10 participants reduced PWV, ranging from − 0.1 to − 2.8 m·s⁻¹, with one participant showing the most significant decrease of − 2.8 m·s⁻¹. Two individuals exhibited small increases of 0.1 and 0.9 m·s⁻¹. In contrast, the LV_chemo_ group (green bars) displayed a consistent reduction in PWV, ranging from − 0.2 to − 4.0 m·s⁻¹. Exceptions included one participant with no change (0.0 m·s⁻¹) and another showing an increase of 1.5 m·s⁻¹ (Supplementary Fig. S2A). Additionally, regarding individual changes in cIMT_max_, all participants in the HVchemo group (red bars) exhibited decreases ranging from − 0.01 to − 0.63 mm (Supplementary Fig. S2B). By contrast, in the LV_chemo_ group (green bars), some participants demonstrated increases up to + 0.28 cm, others experienced substantial decreases, with the most significant reduction reaching − 0.94 mm (Supplementary Fig. S2B).

### Pre–post training changes in secondary vascular outcomes

**Fig. 2:**
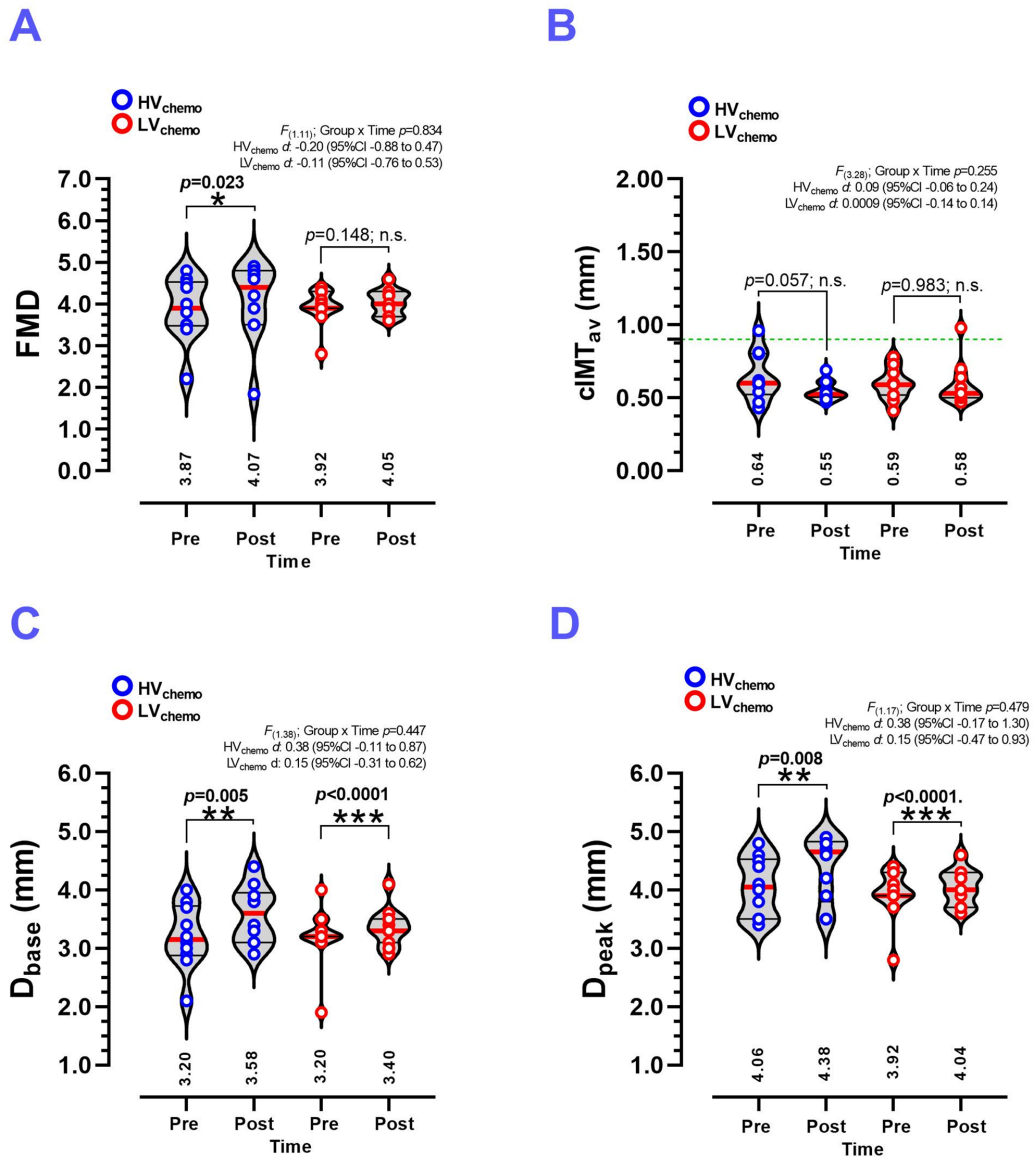
Flow mediated dilation of the brachial artery (**A**), carotid intima-media thickness in *average* (**B**), brachial artery diameters at baseline (**C**) and peak diameter post-5 min of antecubital occlusion (**D**) measured before and after 8 weeks of concurrent training in women breast cancer survivors with history of high-volume and low-volume of chemotherapy treatment. **Groups are described as;** (HV_chemo_) High-volume chemotherapy group, (LV_chemo_) Low-volume chemotherapy group. **Outcomes are described as;** (FMD) Flow mediated dilation, (cIMT_av_) Carotid intima-media thickness *average*, (D_base_) Brachial artery diameter at *baseline*, (D_peak_) *Peak* of the brachial artery diameter after 5 min occlusion of the antecubital artery. (n.s.) No significant modifications. (*) Denotes significant differences between pre vs. post-test at *P* < 0.05. (**) Denotes significant differences between pre vs. post-test at *P* < 0.01. (***) Denotes significant differences between pre vs. post-test at *P* < 0.0001. (*d*) Denotes Cohen d effects size with 95%CI. All bold values denote significant statistical changes/differences at *P* ≤ 0.05

FMD was significantly increased in HV_chemo_ (3.87 ± 0.70 to 4.07 ± 0.94, *p* = 0.023, *d* 0.20) (Fig. 2A), while in the LV_chemo_ group do not report significant statistical changes (Fig. 2A). cIMT_av_ do not elicited changes (Fig. 2B). The HV_chemo_ group significantly increased D_base_ (3.20 ± 0.55 to 3.58 ± 0.49 mm, *p* = 0.005, *d* 0.38) similar than the LV_chemo_ group (3.20 ± 0.50 to 3.36 ± 0.32 mm, *p* < 0.0001, *d* 0.15) (Fig. 2C). Similar findings were observed for D_peak_, where the HV_chemo_ group increased its D_peak_ after CT_HIIT+RT_ (4.06 ± 0.50 to 4.38 ± 0.56 mm, *p* = 0.008, *d* 0.52), similar than LV_chemo_ group (3.92 ± 0.43 to 4.04 ± 0.30 mm, *p* < 0.0001, *d* 0.15) (Fig. 2D).

Individual changes in FMD, cIMT_av_, D_base,_ and D_peak_ are shown in Supplementary Fig. S3. In the HV_chemo_ group, most participants experienced slight positive changes in FMD, ranging from + 0.1% to + 0.6%, except for one subject who showed a decrease (–0.3%). In the LV_chemo_ group, improvements were also seen, with values ranging from + 0.1% to + 0.8%, while one participant showed a slight decrease (–0.2%) (Supplementary Fig. S3A). Supplementary Fig. S3, panel B, illustrates changes in cIMT_av_, where the HV_chemo_ group, comprising most participants, demonstrated reductions from − 0.01 to − 0.23 mm. In the LV_chemo_ group, several participants showed increases in cIMT_av_ (up to + 0.22 mm), While others showed decreases, with the largest reduction being − 0.26 mm, indicating more varied responses (Supplementary Fig. S3B). In the HV_chemo_ group, nearly all participants had small positive changes in D_base_ from + 0.01 to + 0.07 mm, while a few had no change. In the LV_chemo_ group, responses in D_base_ were mixed, with some showing small positive changes (+ 0.01 to + 0.03 mm), but others had reductions, reaching − 0.04 mm (Supplementary Fig. S3C). Additionally, in the HV_chemo_ group, most participants experienced small increases in D_peak_ from + 0.01 to + 0.07 mm, with some unchanged, while in the LV_chemo_ group, improvements ranged from + 0.01 to + 0.08 mm; however, a few participants experienced reductions up to − 0.04 mm (Supplementary Fig. S3D).

### Pre–post training changes in muscle strength outcomes

**Fig. 3:**
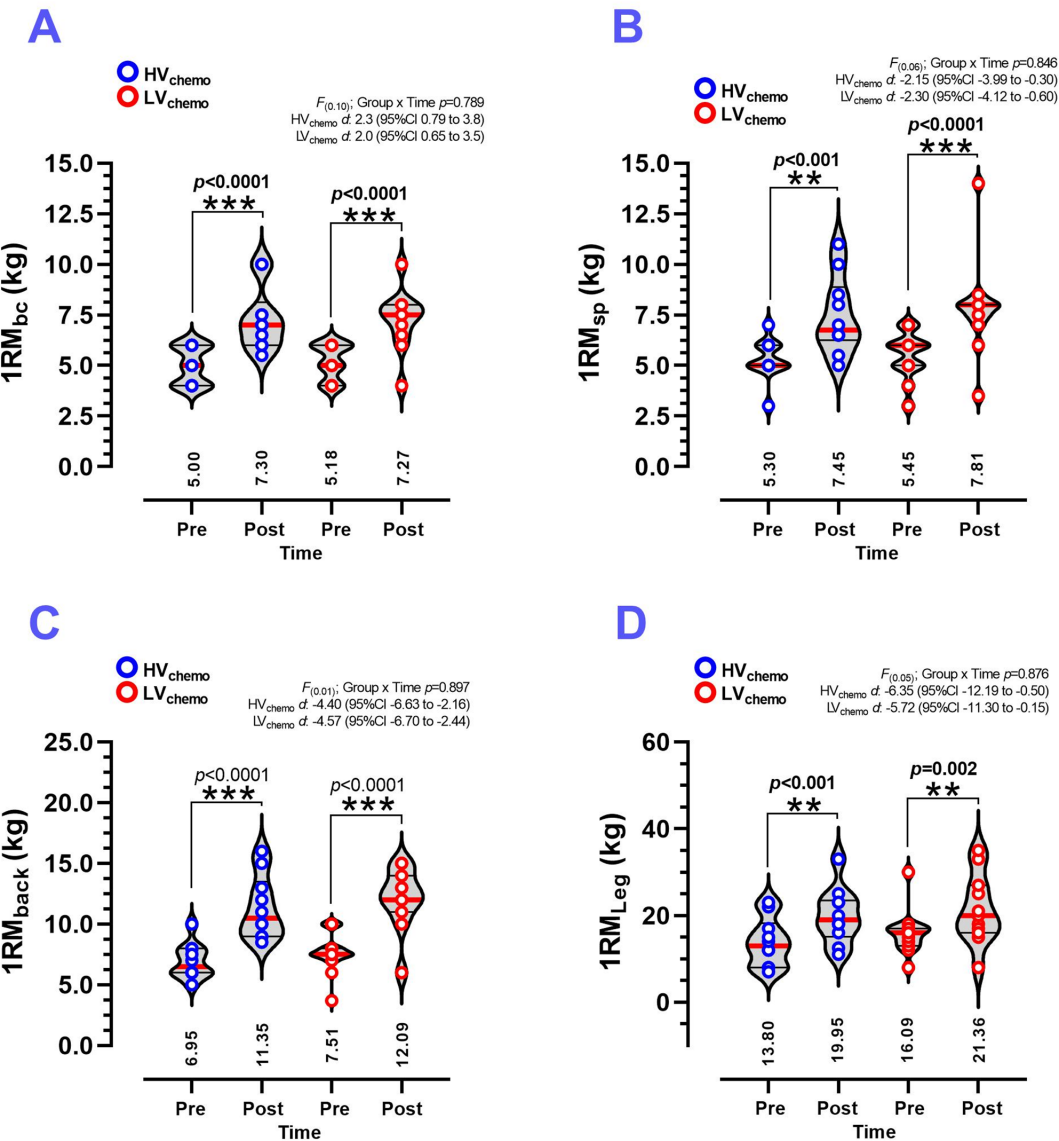
Muscle strength measurements of the biceps curl (**A**), shoulder (**B**), back (**C**), and leg (**D**) measured before and after 8 weeks of concurrent training in women breast cancer survivors with history of high-volume and low-volume of chemotherapy treatment. **Groups are described as;** (HV_chemo_) High-volume chemotherapy group, (LV_chemo_) Low-volume chemotherapy group. **Outcomes are described as;** (1RM_bc_) One maximum repetition strength test of biceps curl, (1RM_sp_) One maximum repetition strength test of shoulder press, (1RM_back_) One maximum repetition strength test of back exercise, and (1RM_Leg_) One maximum repetition strength test of leg-extension. (**) Denotes significant differences between pre vs. post-test at *P* < 0.01. (***) Denotes significant differences between pre vs. post-test at *P* < 0.0001. (*d*) Denotes Cohen d effects size with 95%CI. All bold values denote significant statistical changes/differences at *P* ≤ 0.05

1RM_bc_ after CT_HIIT+RT_ was significantly increased in both HV_chemo_ (5.00 ± 0.94 to 7.30 ± 1.56 kg, *p* < 0.0001, *d* 2.3) and LV_chemo_ groups (5.18 ± 0.87 to 7.27 ± 1.48 kg, *p* < 0.0001, *d* 2.0) (Fig. 3A). 1RM_sp_ after CT_HIIT+RT_ was significantly increased in both HV_chemo_ (5.30 ± 1.05 to 7.45 ± 1.92 kg, *p* < 0.001, *d* 2.15) and LV_chemo_ groups (5.45 ± 1.21 to 7.81 ± 2.48 kg, *p* < 0.0001, *d* 2.30) (Fig. 3B). 1RM_back_ was significantly increased in both HV_chemo_ (6.95 ± 1.46 to 11.35 ± 2.60 kg, *p* < 0.0001, *d* 4.40) and LV_chemo_ groups (7.51 ± 1.73 to 12.09 ± 2.54 kg, *p* < 0.001, *d* 4.57) (Fig. 3E). Similarly, 1RM_Leg_ after intervention was significantly increased in both HV_chemo_ (13.80 ± 5.61 to 19.95 ± 6.44 kg, *p* < 0.001, *d* 6.35) and LV_chemo_ groups (16.09 ± 5.44 to 21.36 ± 8.04 kg, *p* = 0.002, *d* 5.72) (Fig. 3F).

## Discussion

The present study aimed to determine the effects of 8 weeks of concurrent training of CT_HIIT+RT_ on functional (i.e., reported by PWV and FMD) and structural vascular outcomes (i.e., reported by PWV, cIMT_max_ and other secondary outcomes such as cIMT_av_, D_base_, and D_peak_) of breast cancer survivors with a history of high or low exposure to chemo sessions. The present study demonstrates that at least in the subjects with more exposure to chemo sessions, the CT_HIIT+RT_ induces *i*) a reduction in the cIMT_max_ (∆˗0.23 mm) (Fig. 1A) with 9 out of 10 subjects elicited some reduction (range: ˗0.01 to ˗0.63 mm) in the HV_chemo_ group (Supplementary Fig. S1); and *ii*) an increased FMD in the same HV_chemo_ group (∆ +0.20 allometric calculated and [∆+5.1%] in absolute percentage) (Fig. 2A) with 8 out of 10 subjects elicited some reduction (range: +0.06 to + 0.01) (Supplementary Fig. S2), *iii*) significant increases in the baseline (D_base_ ∆ +0.38 mm) and peak diameter after the 5 min occlusion forearm maneuver (D_peak_ ∆ +0.32 mm) in the same HV_chemo_ group (Fig. 2C-D), and *iv*) each group reported increases in CRF (Table 1), muscle strength in biceps, shoulder, back, and leg-extension exercise (Fig. 3A-D). These results were displayed with other benefits in the LV_chemo_ group, who showed improvements in their vascular function by decrease PWV, and increases in D_base_ and D_peak_.

Regarding our initial finding of a ∆˗0.23 mm reduction in cIMT_max_ (Fig. 1B), our data showed structural vascular improvements in the HV_chemo_ group, indicating that the CT_HIIT+RT_ intervention leads to a favorable slowdown of atherosclerotic progression and suggests positive vascular remodeling after training. Notably, it has been previously shown that an 8-week HIIT exercise program did not significantly change the commonly reported cIMT_av_ outcome (∆–0.002 mm) in breast cancer patients undergoing chemo. Still, during the intervention period, the control group showed significant increases in cIMT_av_ [19], indicating that HIIT was effective in maintaining wall thickness in breast cancer patients, thereby helping to reduce cardiotoxicity and the progression of atherosclerotic symptoms in this population [19]. Additionally, the European Society of Hypertension and European Society of Cardiology [24] have stated that cIMT_av_ values > 0.90 mm denote high cardiovascular risk, and our data show that both groups reduced cIMT_max_ below 0.90, resulting in a significant reduction in cardiovascular risk in those patients. At the same time, an increased cIMT_max_ use as an atherosclerotic cardiovascular risk factor has recently been highlighted by Pavanello et al. [25], who considered its association with cIMT_av_ and the increased triglyceride-glucose ratio. Thus, these findings support the notion that short–term CT_HIIT+RT_ offer comparable or superior structural vascular adaptations that could play a key role in arterial vascular remodeling compared to more prolonged (i.e., in terms of session duration and exercise volume) and intense exercise programs to decrease the cardiovascular risk, particularly in women with breast cancer who received more chemo volume.

Regarding our second result, which shows an (∆+0.20 allometric scaling calculated but an absolute percentage [∆+5.16%]) increase in FMD in the HV_chemo_ group, it is important to note that the inter-individual variability data provided (Supplementary Fig. S1) indicates that, on a subject-by-subject basis, 8 out of 10 subjects showed increased FMD, while 6 out of 11 subjects in the LV_chemo_ group did so. Lee et al. [19] in a similar volume study, it was reported that 8 weeks of HIIT (60 s x 7 intervals, with 2 min active recovery at 90% of peak power output), FMD was increased from 12.6 to 16.9% (∆+4.3%) in breast cancer patients under doxorubicin and cyclophosphamide chemo treatment. In adults without cancer, a meta-analysis from Brislane et al. [26] including (*n* = 182) subjects summarized that MICT increased FMD in postmenopausal women with higher blood pressure, and the effects were greater in those with higher resting blood pressure and the greatest CRF (i.e., by maximum oxygen consumption) reported major FMD increases. Current evidence from Siripanya et al. [27] in breast cancer patients (from 40 to 60 y) under anthracycline chemotherapy, it was reported that MICT (for 30 min/session, 3 times/week for 12 weeks) plus a meditation strategy increased FMD, CRF by VO2_peak_, but no PWV changes. Thus, our results are accordance with previous evidence [19] and suggest that shorter CT_HIIT+RT_ interventions integrating upper and lower limb RT exercises, as well as abbreviated CRF stimuli as short HIIT protocols (60 s x 5 intervals) plus low RT exercise in upper limb can be sufficient stimuli to increase brachial artery function by increasing FMD promoting thus lower cardiotoxicity in breast cancer survivors independent of their different chemo volume history.

About our third result of increased brachial artery D_base_ ∆ +0.38 mm and D_peak_ ∆ +0.32 mm after reactive hyperemia in the HV_chemo_ group (Fig. 2C-D), along with this, in the study of Lee et al. [19] in breast cancer patients, 8 weeks of HIIT exercise increased the D_base_ from 3.62 to 3.69 (∆+0.07 mm), with the brachial artery thickness also slightly growing, suggesting that potentially lower-limb HIIT exercise could also confer upper-limb or systemic vascular benefits in these cohorts. On the other hand, Green et al. [18] developed a MICT exercise program by treadmill walking and cycling for 24 weeks, revealed a nonsignificant increase in D_base_, but a significant decrease in both popliteal and brachial artery wall thickness. Although we did not measure lower-limb artery thickness in the brachial artery, considering previous evidence [18], and the detrimental process that breast cancer surgery implies in the upper limb arteries, there is a need for future studies exploring the physiological vascular adaptations phenomenon after exercise training interventions.

Among our secondary outcomes, the average PWV decrease of (∆˗1.64 m·s^− 1^) in the LV_chemo_ group (Fig. 1A), which unfortunately includes participants who did not decrease by ≤ 10 m·s^− 1^ (the cut-off point for high cardiovascular risk), may also be relevant. Jones et al. [28] reported that PWV decreases of (∆–0.3 m·s^− 1^) after 12 weeks of CT_MICT+RT_, whereas our results were superior but also of lower volume (8 vs. 12 weeks of CT). Additionally, previous findings from our laboratory have been shown that 6 weeks of CT_HIIT+RT_ increases FMD (∆+7.7%), and decreases PWV after 6 (∆–1.2 m·s^− 1^) [22], and 12 weeks (∆–0.5 m·s^− 1^) of multiple exercise modalities [16] or previous reductions in the cIMT_max_ (∆−0.10 mm) [23] in hypertensive patients. These results suggest that CT_HIIT+RT_ exercise regimes can be effective in interventions between 8 and 12 weeks for decreasing cardiotoxicity symptoms in breast cancer survivors.

Regarding CRF (Table 1) and muscle strength results (Fig. 3A-D). A current literature review from Madeira et al. [29] which assessed different CT regimes, from 4 to 36 weeks of duration, including CT_MICT+RT_ and CT_HIIT+RT,_ in women breast cancer survivors, showed that only one study [Mijwel et al. [30] 16 weeks of CT_HIIT+RT_ (HIIT; twice a week, 60 min per session by bike, and RT; 2–3 sets of 8–12 repetitions, at 70–80% of 1RM) increased VO2_peak_, and improved several cancer–related fatigue outcomes. We observed significant increases in VO2_peak_ in the HV_chemo_ (∆+4.9 mL·kg·min^− 1^) and LV_chemo_ groups (∆+3.9 mL·kg·min^− 1^), with both groups reporting benefits. A recent study of Scott et al. [31] in (*n* = 158) breast cancer patients revealed that 2 weeks of chemo treatment decreased VO2_peak_ (∆~1.5 mL·kg·min^− 1^)^,^ but under CT exercise or usual care physical activity recommendations, CT increased this outcome (∆+3.5 mL·kg·min^− 1^) that was almost twice vs. usual care but importantly displayed with systolic blood pressure reductions of (∆˗5.2 mmHg).

On the other hand, muscle strength was increased in each HV_chemo_ and LV_chemo_ groups (Fig. 3), where relevant institutions as the ACSM have promoted the assessment and the inclusion of muscle strength components into an oncology exercise program [32] considering strong evidence to improve cancer-related health such as anxiety, depressive symptoms, fatigue, physical functioning, and health-related quality of life, suggesting RT intensities between 60 and 80% of 1RM. Previous evidence has also shown that moderate-to-high–intensity RT is more effective for preventing lymphedema by reducing extracellular water retention and preventing fluid accumulation than low–intensity RT [17]. However, in the present study, we show that lower RT intensities (20 to 50% of 1RM) as part of a CT_HIIT+RT_ regime also increase muscle strength in three upper- and one lower-limb muscle groups, which is relevant to maintaining functionality and metabolic control.

In addition, previously, Lee et al. [33] reported that 16 weeks of MICT and RT three–weekly in sedentary breast cancer patients (stage I to III), reduced the Framingham Risk Score, summarizing that exercise was effective to mitigate the adverse effects of chemo on the cardiovascular system, reducing the risk of cardiovascular disease, being our results in coherence with this previous literature. A recent review article from Briggs et al. [34] suggests that the most appropriate interventions for breast cancer patients are multimodal, personalized, where not only exercise, but also stress, cognitive, smoking and alcohol cessation, immune–nutrition, and education, play a role in the rehabilitation of cancer survivors. From here, and as our study consisted only of testing a particular CT_HIIT+RT_ protocol, it is relevant for breast cancer rehabilitation to consider the key components for more successful programs.

Part of the vascular remodeling from aerobic exercise includes enhanced nitric oxide production through the activation of endothelial nitric oxide synthase, which reduces oxidative stress by decreasing nicotinamide adenine dinucleotide phosphate oxidase activity, thereby lowering reactive oxygen species production. Improves nitric oxide bioavailability, reducing vasoconstriction and improving endothelial function, preventing fibrosis, and improving the perivascular adipose tissue function [12]. Also, exercise promotes epinephrine and norepinephrine as monoaminergic factors that control breast cancer and cell viability by tumor suppression [35].

### Limitations and strengths

As limitations, we recognize that *i*) the 1RM test were applied bilaterally taking care about potential reduction in muscle strength from the breast surgery side in each patient (see Table 1 characteristics), that could not revealed the maximum real strength in each arm, however we educated verbally about this test to each patient, *ii*) we calculated the scaling allometric FMD that by the accepted formulae of D_base_ and D_peak_^(Slope)^ and not using an automatic vascular software, according with previous studies under brachial artery diameter changes after exercise interventions [36], however, we also show FMD in absolute % (HV_chemo_ ∆+5.16 and LV_chemo_ ∆+3.31%), *iii*) when FMD_allo_ calculation is carried out, other methodological issues are also relevant to consider, e.g., when the protocol of adding 50 mmHg occlusion over the baseline SBP at pre-test, but in post-test time SBP is decreased from pre-test it promotes a lower stimuli for brachial artery between times in which shear rate can be useful to manage, *iv*) there is no consensus about what chemo is of ‘higher or ‘low volume’, where by the present study we propose to divide in the total number of chemo sessions received by each participant, *v*) baseline PWV values were different between HV_chemo_ vs. LV_chemo_ groups, however, both groups were almost similarly at borderline (9 to 10 m·s^− 1^) or in high risk (~ 10 m·s^− 1^) in this arterial stiffness marker, *vi*) subjects with a history of discontinuing chemo treatment due to chemotherapy-induced cardiotoxicity did not participate in this study, *vii*) the present study did not aimed to explore further into the types or chemo scheme received, beyond describing the quantities (i.e., volume) of chemo sessions declared within each participant’s individual history, and *viii*) our chosen and applied statistical linear model was not compared with statistical mixed models; therefore, the statistical power and coefficient estimates could potentially differ from some results [37]. Some strengths include that *i*) we used the standard FMD technique with validated protocol including the D_base_ and D_peak_ that were functional results and *ii*) PWV equipment, cIMT_av_, cIMT_max_, D_base_ and D_peak_ of the brachial artery as structural vascular markers.

## Conclusion

Eight weeks of CT_HIIT+RT_ in women breast cancer survivors who received higher chemotherapy volume reduces cIMT_max_, but increases FMD, D_base_, D_peak_, cardiorespiratory fitness, and muscle strength. The vascular improvements are only partially observed in subjects with lower chemotherapy volume.

## Methods

### Population and study design

This is a preliminary report from the Onco–Vascular Exer Study, an experimental study involving twenty–one adult females, cancer survivors from diverse socio-economic groups linked to a university community and staff. The research team attended breast cancer support group meetings where the study was advertised. Then, the team received interest from several breast cancer patients by e-mail and phone who were active participants in cancer survivors’ social groups. All interested breast cancer survivors were invited for a personal interview and screening. Once interested subjects agreed to participate in the study, they signed the informed consent. General and specific cancer treatment information, along with their clinical history, was part of the study screening. Participants were assigned to one of two exercise groups based on their previously registered chemo dosage: the high-volume (HV_chemo_) or low–volume chemo (LV_chemo_) group, and agreed to undergo 8 weeks of CT_HIIT+RT with_ three sessions per week. The study is registered under ClinicalTrials.gov (NCT06766903) and was approved by the Ethical Committee of the Universidad Andres Bello (Chile) by Approval 005/2024 of April 12th. The study was conducted in accordance with the Declaration of Helsinki for human studies.

The Onco–Vascular Exer Study study included the following inclusion criteria: *i*) breast cancer diagnosis, *ii*) receiving at least one session of chemo, *iii*) a body mass index (BMI) between 18.6 and 39.9 kg/m^2^, *iii*) age between 30 and 75 years. Exclusion criteria included; *i*) history of abnormal ECG such as arrhythmias or other electrical cardiac abnormalities, *ii*) controlled cancer hormonal therapy, *iii*) controlled comorbidities therapy for prediabetes or diabetes, prehypertension hypertension, metabolic syndrome, fatty liver disease or hypercholesterolemia [13], *iv*) declare all pharmacotherapy uses under emergencies or SOS to manage pain such as morphine patches, morphine droplets, *v*) diagnosis or history of cardio–vascular disease (e.g., other than controlled hypertension), including vasculopathy, *vi*) history of uncontrolled stage 3 hypertension or hypertensive crisis, *vii*) diabetes–related complications such as varicose ulcers, nephropathies, *viii*) muscle skeletal abnormalities (e.g., knee or hip osteoarthritis, non-cancer-related muscle pain). *ix*) use of pharmacotherapy influencing weight loss, *x*) being active or under exercise training programs or involved in exercise program the past three months, *xi*) respiratory diseases (e.g., chronic obstructive disease), *xii*) kidney disease, *xiii*) pregnancy, *xiv*) smoking behavior or dependence on other substances.

**Fig. 4:**
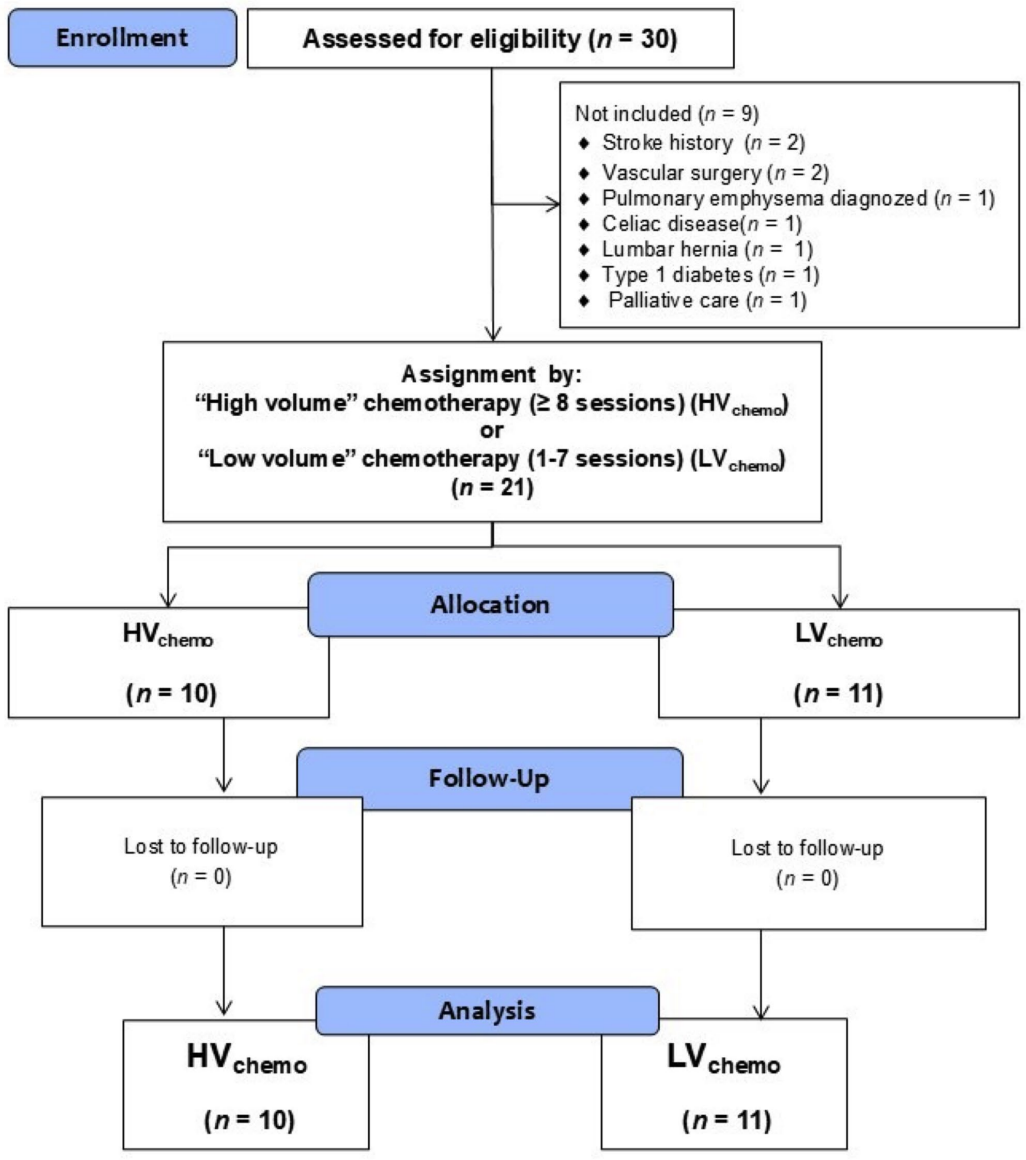
Study design. Groups are describes as; (HV_chemo_) High volume chemotherapy. (LV_chemo_) Low volume chemotherapy sessions

The final sample size included the high–volume (HV_chemo_; *n* = 10) and low–volume chemo (LV_chemo_; *n* = 11) groups. The study design can be seen in Fig. 4.

### High and low history of chemotherapy volume categorization

To categorize both HV_chemo_ and LV_chemo_, during the personal interview, each subject was screened about their medical history and socio–demographic information. Considering that the maximum chemo sessions declared by a participant was of 16, from the pre-test, all those who claimed to have been intervened for ≥8 chemo sessions were categorized as high–volume of chemo treatment (HV_chemo_), and on the other hand, those who declared between 1 and 7 chemo sessions were categorized as low–volume chemo sessions (LV_chemo_).

### Anthropometric and body composition

Weight and body composition (body fat, %; skeletal muscle mass, kg; and free fat mass, kg) were measured by bio–impedance (InBody270™, tetrapolar 8-point tactile electrode system, model BPM040S12F07, Biospace, Inc., Seoul, Korea) with a 0.1 kg precision. Height (m) was measured with a stadiometer (HEALTH O METER™ Professional, Sunbeam Products, Inc., Chicago, IL, United States). Waist circumference was measured using an inextensible tape (SECA™, United States). Body mass index (BMI) was calculated using both weight divided by the square of height [38]. These measurements were performed in the morning between 9 and 13 h at the Universidad Andres Bello Laboratory.

### Blood pressure measurement

Blood pressure (BP) was measured following the European Society of Hypertension and European Society of Cardiology guideline [24]. After a 10-minute rest period, three measurements were applied in the left forearm using an automatic blood pressure monitor (OMRON™ HEM 7114, Japan). When a difference ≥ 10 points was observed between these measurements, a new measurement was applied to corroborate the data. Systolic (SBP) and diastolic (DBP) blood pressure values were obtained and defined as follows: <129/85 mmHg as normotension, 130 to 139/90 mmHg as high–normal blood pressure, and ≥ 140/90 mmHg as hypertension. The medical and pharmacological characteristics of patients can be seen in Table 2.

**Table 2:** Characteristics of participants

Outcomes	HV_chemo_	LV_chemo_
*n* **=**	10	11
Time from diagnosed (y)	5.0 ± 5.4	8.6 ± 4.8
Chemotherapy (sessions)	11.3 ± 3.0	3.0 ± 2.5
Radiation (sessions)	14.7 ± 8.6	16.0 ± 10.5
Children (n°)	1.9 ± 1.2	2.0 ± 0.6
*Surgical information*		
Affected breast (right)	3/10	6/11
Affected breast (left)	5/10	4/11
Affected breast (both)	2/10	1/11
Lymphedema	1/10	0/11
*Hormonal therapy*	6/10	5/11
Tamoxifen	3/10	4/11
Anastrozole	3/10	1/11
*Statins*		
Atorvastatin	1/10	1/11
Rosuvastatin	1/10	2/11
*Blood pressure*		
Hypertensive	1/10	4/11
Diabetes	1/10	1/11
Losartan	2/10	0/11
Olmesartan	0/10	1/11
Hydrochlorothiazide	0/10	1/11
*Endocrine/metabolism*		
Eutirox	1/10	0/11
*Pain*		
^SOS^Paracetamol		
^SOS^fentanyl patch	0/10	1/11
^SOS^drops of morphine	0/10	1/11
*Other*		
Omeprazol	1/10	1/11
Esomeprazole	0/10	1/11
^SOS^Aspirin	0/10	1/10
C Vitamin	5/10	0/11
B Vitamin	1/0	0/11
Zinc	2/10	0/11
Magnesium citrate	5/0	0/11
^SOS^Zopliclone	1/10	0/11
^SOS^Pregabalin	1/10	1/11
^SOS^Duloxetin	1/10	1/11
^SOS^Lamotrigine	1/10	1/11

Data are shown as mean and ± SD to continuous outcomes, or as proportion. (SOS) Denotes medication prescribed under emergencies or occasional mode for patients.

### Flow mediated dilation (FMD)

After 10 min of resting, all participants had their blood pressure evaluated (OMRON™ HEM 7114, Japan). All explorations were carried out by using an ultrasound equipment (GE™, Model LOGIQ-E PRO, Milwaukee, United States) with a 7 − 12 MHz linear–array transducer. FMD calculation was developed in six phases; first, each participant was placed in supine position for 20 min on a medical bed, with the arm in abduction 90°, a blood pressure cuff (RIESTER model ri-san™, Jungingen, Germany) was positioned on the forearm and the ultrasound transducer (i.e., 1–3 cm proximal to the antecubital fossa on a longitudinal plane) was installed in the brachial artery using an adjustable mechanical arm precision holder (EDI™, Progetti e Sviluppo, Italy), to maintain stable the transducer position, and increase evaluator standardization. Thus, baseline diameter (D_base_) of the brachial artery images (in B and Doppler-mode) was obtained (i.e., during 30 to 60 s) and stored to after in off-mode develop the D_base_ measurement (i.e., 2 to 4 diameter measurements and the average was registered). Second, the blood pressure cuff of the forearm was inflated at 50 mmHg over the systolic blood pressure of resting, starting the 5 min of the forearm occlusion to induce reactive hyperaemia [39]. Third, immediately after cuff deflation, every 10 s for 120 s (12 images) were obtained and stored to determine the D_peak_ measurement (i.e., 2 to 4 diameter measurements and the average was registered) in each image. Fourth; FMD obtained from the formulae: $\:\mathrm{F}\mathrm{M}\mathrm{D}\:\left(\mathrm{\%}\right)=\frac{\left[\left(\mathrm{D}\mathrm{p}\mathrm{e}\mathrm{a}\mathrm{k}\:-D\mathrm{b}\mathrm{a}\mathrm{s}\mathrm{e}\right)\right]*100}{\mathrm{D}\mathrm{b}\mathrm{a}\mathrm{s}\mathrm{e}}$ Fifth; After this procedure, and considering the changes in the diameter of the brachial artery due to the CT_HIIT+RT_, each FMD data from each participant was processed to calculate by the *allometric scaling* flow-mediated dilation (FMD_allo_) reported previously from Atkinson et al. [36] and recently highlighted recently by Gomez et al. [40], which considers the following steps; (a) calculation of D_peak_ and D_base_ in (mm), (b) logarithmic transformation of both D_peak_ and D_base_ (Log_D_base_ and Log_D_peak_), (c) calculation of the ‘slope’ (Slope) value obtained from the linear regression between both Log_D_base_ and Log_D_peak_, and finally, (d) the application of the formulae proposed: FMD = D_peak_ / D_base_^(Slope)^. Sixth; Once pre and post FMD_allo_ values were obtained, we calculate the absolute percentage (%) of modification by group. Some specific considerations include that all measurements were developed in the brachial artery of the more comfortable side of the patient (e.g., if the patient had a breast surgery history, the manoeuvre was applied in the contralateral arm).

### Arterial stiffness

Arterial stiffness was measured by aortic PWV by a digital cuff equipment in the brachial artery for inflation–deflation reporting in (m·s^− 1^) (Arteriograph, TENSIOMED™, Hungary) [41]. After a 20–minute rest in supine position, PWV was measured using a laptop and the (Arteriograph Software™ v.1.9.9.2) for PWV analyses, inflating/deflating the cuff twice for a duration of ~ 5 min. The PWV data was obtained from a PDF report and was extracted from the software. A schematic image of FMD and PWV can be seen in Supplementary Figure S4.

### Carotid intima–media thickness (cIMT) measurement

We measure both cIMT_av_ and cIMT_max_, using the same ultrasound with 7 − 12 MHz linear–array transducer (GE Medical Systems, Model LOGIQ-E PRO, Milwaukee, United States). All subjects were in supine position for 20 min. After carotid bulb identification, an image was acquired in B-mode in the correct longitudinal orientation of the common carotid artery using an automatic image capture function that detects both cIMT_av_ and cIMT_max_. The scan was focused 1 to 2 cm from the carotid bifurcation on the far wall. Using GE Healthcare’s automated detection software, the ultrasound identifies the carotid intima-media region, where the characteristic bright–dark–bright pattern corresponds to the cIMT_av_. Thus, the ultrasound equipment delivers the cIMT_av_ in numeric data, but at the same time calculates the maximum height within this length (10 mm) of the cIMT_av_ measurement, which corresponds to the cIMT_max_. This automated method yields reliable measurements of both cIMT_av_ and cIMT_max_ and minimizes the subjectivity inherent to manual assessments. The ultrasound recorded the image, which was later analyzed offline. All measurements were recorded at the end–diastolic stage [42].

### One repetition maximum (1RM) strength test

All subjects were instructed to rest the day before. Under the researcher’s instructions, each subject used dumbbells to perform the tests. Once they observed examples and were trained on the execution of proper lifting techniques, each participant made three attempts (with 1 1-minute rest period each) to achieve their highest 1RM. Thus, the order for the 1RM test was leg flexion–extension (1RM_Leg_) with a leg-extension quadriceps bench machine (PRIM™ 503291, Spain), followed by upper back (1RM_back_), shoulders (1RM_sp_), and biceps (1RM_bc_) tested with dumbbells.

### Cardiorespiratory fitness (CRF)

**Fig. 5:**
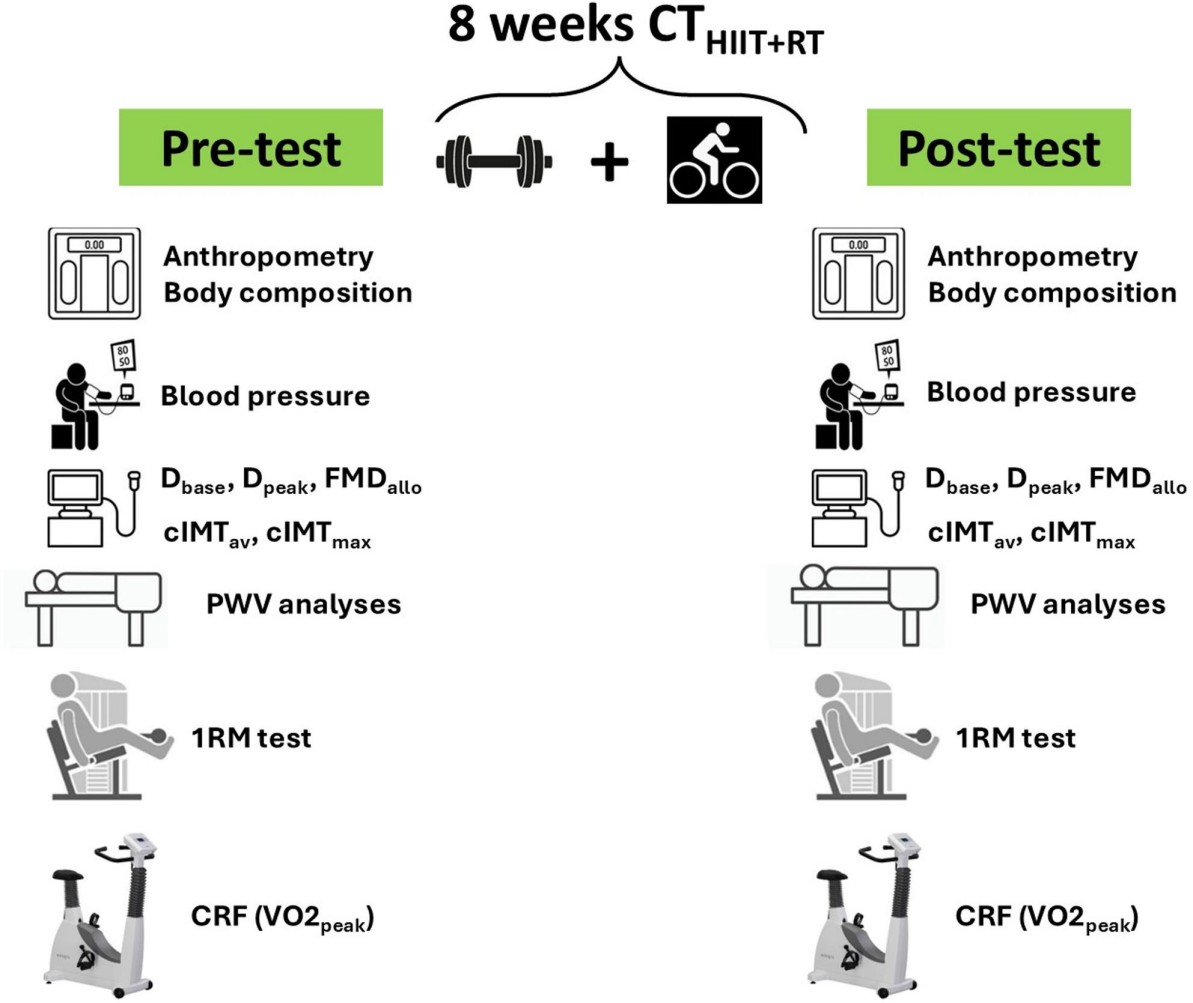
Study protocol of 8 weeks of exercise intervention period of concurrent training including high–intensity interval plus resistance training, showing pre and post–test measurements. Outcomes are described as: (D_base_) Brachial artery diameter baseline, (D_peak_) Peak of the brachial artery diameter after 5 min occlusion of the forearm, (FMD) Flow-mediated dilation, (cIMT_av_) Carotid intima media thickness *average*, (cIMT_max_) Carotid intima media thickness *maximum*, (PWV) pulse wave velocity, (1RM) One-maximum repetition strength test, (CRF) Cardiorespiratory fitness test, and (VO2_peak_) Peak oxygen consumption

CRF was assessed using the modified Astrand exercise test, which estimates peak oxygen consumption (VO2_peak_) and obtains peak heart rate (HR_peak_) [43, 44]. Each participant was instructed to be more active 7 days before the test (i.e., to avoid plenty of sedentarism in daily activities) but not doing high–intensity efforts. Similarly, consume carbohydrates at least 24 h before the test, stay hydrated with water, and avoid caffeine on the day of the test. During the test, heart rate was continuously measured during each stage by a heart rate monitor on the left wrist (A370, Polar™, Kempele, Finland) [45]. Using a cycle-ergometer (Ergoselect 200, ERGOLINE™, Germany), the subjects started the test with 2 min of warm–up cycling without load, and then exercise intensity was increased by 25 watts every 2 min until volitional exhaustion. The overall study protocol and measurements are shown in Fig. 5.

### Concurrent training of high–intensity interval plus resistance training

The CT_HIIT+RT_ was 3 times per week, with 48–72 h between sessions. Each CT_HIIT+RT_ session included, firstly, the HIIT stage using stationary bikes, and then the RT section using free weights, individually accommodated to each subject. For HIIT, each subject developed 5 repetitions of 60 s during weeks 1 to 4, and 7 repetitions of 60 s during weeks 5 to 8, at an intensity between 80 and 100% of HR_peak_ and with resting periods until HR returned to ≤ 70% HR_peak_, always controlled individually by heart rate monitors (Model A370, Polar™, Finland) on an upright stationary bicycles (Impulse™, model PS 300, Sparta, Chile) [46]. After 3 to 5–min cool–down, the RT section started, and participants completed 5 sets of 60 s of resistant exercises as follows; (1) biceps curl [2 sets], (2) shoulder press [2 sets], and (3) upper back exercise [1 set], performed at 20 to 50% of 1RM (weeks 1–4) plus step exercise and flexing/extending calf muscles, both (60 s x 2) from weeks 5–8, and with a resting period between sets until a modified Borg scale rating of 1 to 3, out of 10 points. Total workout session last for ~ 30–40 min and both HIIT and RT followed the ACSM guidelines [47]. To regulate each HIIT and RT intensity, each subject received accommodation of bike–load session–by–session (to HIIT; according with the 80% to 100% of HR_peak_), and at level of muscle strength to RT (perceived muscle exertion 7 to 10 points of the modifies Borg 1–10 scale) to each participant maintain the appropriate individual exercise–intensity. In the first 4 weeks, the exercise order was CT_HIIT+RT_, and in the last 4 weeks, the order was CT_RT+HIIT_ to reduce potential order effects. Blood pressure was evaluated before and after each exercise session; in addition, we included 10 min before exercise and another 10 min after the session for warm-up and cool-down periods, respectively, totaling an estimated ~ 50-minute time investment per session for each participant. When some cases reported muscle/joint pain or inflammation, the muscle/joint involved was not exercised to favor recovery and self-care, and verbal tips were suggested to use ice or cold water during the familiarization period. The characteristics of the CT_HIIT+RT_ exercise intervention are shown in Supplementary Table S1.

### Statistical analysis

Data are presented as the mean ± standard deviation (SD). Normality and homoscedasticity assumptions were tested using Shapiro–Wilk tests. To test baseline differences, one-way ANOVA was carried out. Repeated measures by 2-way ANOVA were carried out to test Group x Time adaptations, with Sidak post-hoc. Cohen’s *d* effect size (< 0.20 = negligible, 0.20–0.49 = small, 0.50–0.79 = moderate, ≥ 0.80 = large) for interactions that showed statistically significant [48]. These tests were developed using Grap Pad Prism 8.0 software (Graph Pad software, San Diego, CA, United States). We also calculated the pre-post-test delta changes modified in each outcome (∆), to show in vascular outcomes (PWV, cIMT_max_, FMD, cIMT_av_, D_base_ and D_peak_) the inter-individual variability subject-by-subject. A mixed-model test was applied to check additional interactions (Group x blood pressure therapy, Group x Age), by using SPSS for Windows, program version 23.0 (SPSS Inc., Chicago, IL, USA). The alpha level was fixed at (*P* ≤ 0.05) for all statistical significance. 

## Supplementary Information

Below is the link to the electronic supplementary material.

Supplementary Material 1

## Data Availability

Data will be made available upon reasonable request.
