# Supplementary material for: Concurrent high-intensity interval plus resistance training improves vascular health in breast cancer survivors with high chemotherapy exposure
Source: Biol Res. 2026 Feb 23;59:20. doi: 10.1186/s40659-026-00677-y (PMC13036957; doi:10.1186/s40659-026-00677-y)
Supplement: Supplementary file 1 — Supplementary Material 1 [file 40659_2026_677_MOESM1_ESM.docx]

**Suplementary results**

**Concurrent high-intensity interval plus resistance training improves vascular health in breast cancer survivors with high chemotherapy exposure**

running title: Concurrent training effects on breast cancer

Cristian Álvarez^1^, Carolina Fuentes^2^, Cristóbal Durán-Marín^3^, Pedro Delgado-Floody^4^, Gabriel Rojas-Rojas^1^, Manuel Gomez^5^, Alvaro Gurovich^6^, David C. Andrade^7*^

^1^ Exercise and Rehabilitation Sciences Institute, School of Physical Therapy, Faculty of Rehabilitation Sciences, Universidad Andres Bello, Santiago, 7591538, Chile.

^2^ School of Kinesiology, Universidad Andres Bello, Santiago, Chile.

^3^ Exercise and Rehabilitation Sciences Institute, Doctorate in Rehabilitation Science Program, Faculty of Rehabilitation Sciences, Universidad Andres Bello, Santiago, Chile.

^4^ Department of Physical Education, Sport and Recreation, Universidad de La Frontera, Temuco, Chile.

^5^ Clinical Applied Physiology Laboratory, College of Health Sciences, The University of Texas at El Paso, El Paso, TX 79968, USA.

^6^ Department of Physical Therapy and Movement Sciences, College of Health Sciences, The University of Texas at El Paso, El Paso, TX, United States.

^7^ Exercise Applied Physiology Laboratory, Centro de Investigación en Fisiología y Medicina de Altura (FIMEDALT), Departamento Biomédico, Facultad de Ciencias de la Salud, Universidad de Antofagasta, Antofagasta, Chile.

*Corresponding author: David C. Andrade, Ph.D.

https://orcid.org/0000-0003-2006-1444

Exercise Applied Physiology Laboratory

Centro de Investigación en Fisiología y Medicina de Altura

Departamento Biomédico

Facultad de Ciencias de la Salud

Universidad de Antofagasta

Av. Universidad de Antofagasta #02800 Antofagasta, Chile

Tel: +56 9 88928666

E-mail: [david.andrade@uantof.cl](mailto:david.andrade@uantof.cl)

[dcandrade@uc.cl](mailto:dcandrade@uc.cl)

**SUPPLEMENTARY TABLES**

| **Supplementary table S1.** Characteristics of the concurrent training progression. | | | | | | | | |
| --- | --- | --- | --- | --- | --- | --- | --- | --- |
|  | **“Familiarization period”** | | | | | | **Complete protocol** | |
|  | **Week 1** | | | **Week 2** | | | **Weeks 3-4** | **Weeks 5-8** |
| Session Number | **1** | **2** | **3** | **4** | **5** | **6** | **7-12** | **13-24** |
| **HIIT** | 15 s x 2 | 15 s x 4 | 30 s x 3 | 30 s x 5 | 45 s x 4 | 60 s x 5 | 60 s x 5 | 60 s x 7 |
| Intensity (beats/min) | 80-100% HR_peak_ (Individually adapted session by session) | | | | | | |  |
| Recovery criteria | Complete resting ≤70% Heart rate | | | | | | |  |
| **RT** | 3 Exercises  -Back:  15 s x 1  -Biceps:  15 s x 1  -Shoulder:  15 s x 1 | 3 Exercises  -Back:  15 s x 2  -Biceps:  15 s x 2  -Shoulder:  15 s x 2 | 3 Exercises  -Back:  30 s x 2  -Biceps:  30 s x 2  -Shoulder:  30 s x 2 | 3 Exercises  -Back:  30 s x 2  -Biceps:  30 s x 2  -Shoulder:  30 s x 2 | 3 Exercises  -Back:  45 s x 2  -Biceps:  45 s x 2  -Shoulder:  45 s x 2 | 3 Exercises  -Back:  45 s x 2  -Biceps:  45 s x 2  -Shoulder:  45 s x 2 | 3 Exercises  -Back:  60 s x 2  -Biceps:  60 s x 2  -Shoulder:  60 s x 2 | 5 Exercises  -Step  60 s x 2  -FEG  60 s x 2  -Back:  60 s x 2  - Biceps:  60 s x 2  -Shoulder:  60 s x 2 |
| Intensity (1RM%/Borg) | 20-50% 1RM / 8-10 points of Borg (1-10 points) | | | | | | | |
| Recovery criteria | ≤3 points Borg (1-10 points) | | | | | | | |
| (HIIT) High-intensity interval training, (RT) Resistance training. (HR_peak_) Heart rate peak, (1RM) One–maximum repetition strength test. (FEG) Flexion-extension gastrocnemius. | | | | | | | | |

**SUPPLEMENTARY FIGURES**

**Supplementary Figure S1**


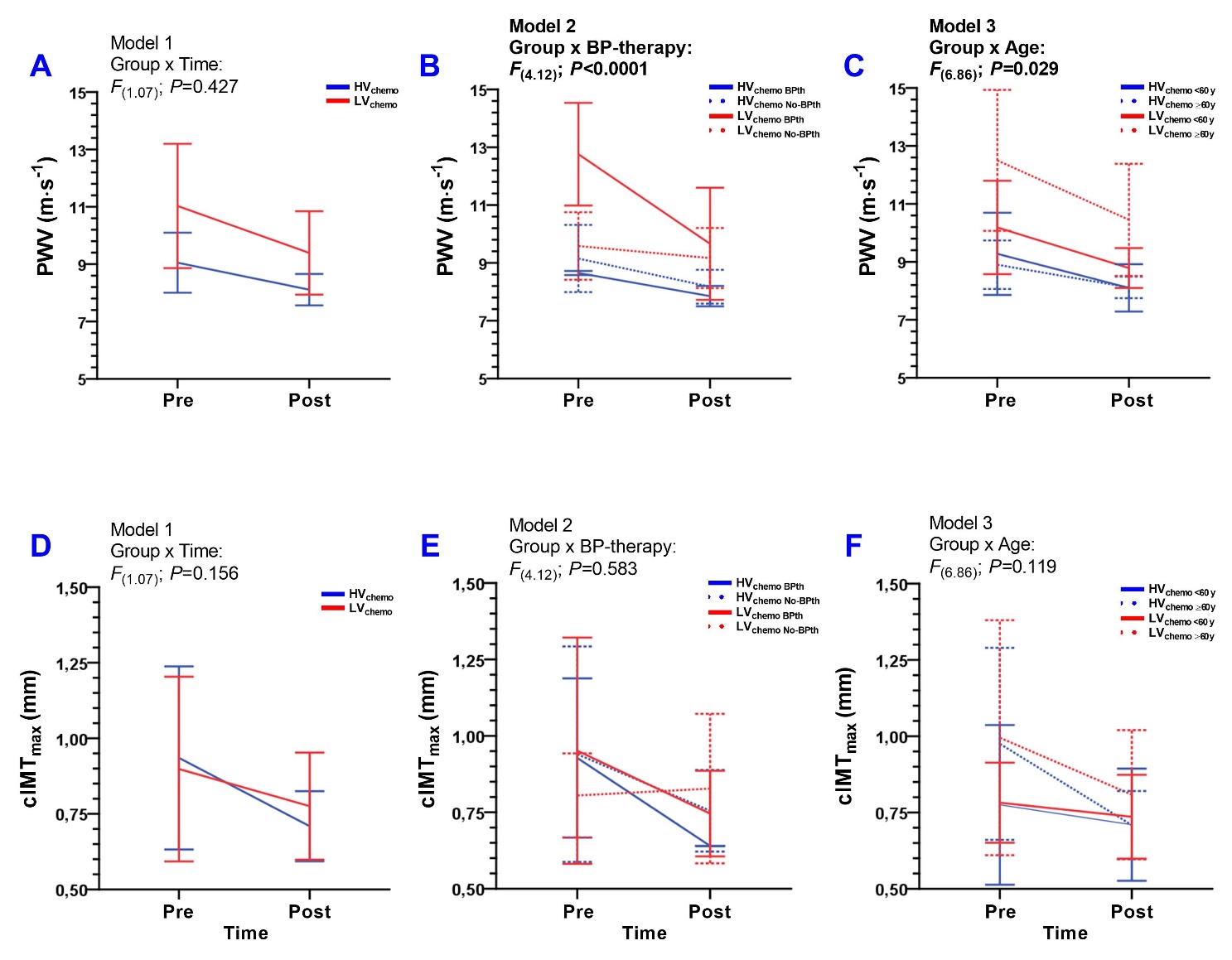


**Supplementary Figure S1.** Interaction of group x time (A, D; Pre vs. Post-test), group x blood pressure therapy (B, E; Blood pressure therapy uses vs. No Blood pressure therapy use) and group x age (C, F; <60 years vs. ≥60 years old) in breast cancer survivor’s participants of 8 weeks of concurrent training. **Groups are described as;** (Panel A and D; [HV_chemo_] High-volume chemotherapy group, (LV_chemo_) Low-volume chemotherapy group, (Panel B and E; [HV_chemo_ BPth] High-volume chemotherapy using blood pressure therapy, [HV_chemo_ No-BPth] High-volume chemotherapy group not using blood pressure therapy), and ([LV_chemo_ BPth] Low-volume chemotherapy using blood pressure therapy, and [LV_chemo_ No-BPth] Low-volume chemotherapy group not using blood pressure therapy), and (Panel C and F; [HV_chemo_ <60 y] High-volume chemotherapy group participants <60 years old, [HV_chemo_ ≥60 y] High-volume chemotherapy group participants ≥60 years old, [LV_chemo_ <60 y] Low-volume chemotherapy group participants <60 years old, and [LV_chemo_ ≥60 y] Low-volume chemotherapy group participants ≥60 years old. **Outcomes are described as;** (PWV) Pulse wave velocity, and (cIMT_max_) Carotid intima-media thickness *maximum*. Bold values denote significant interaction at *P*<0.05.

**Supplementary Figure S2**


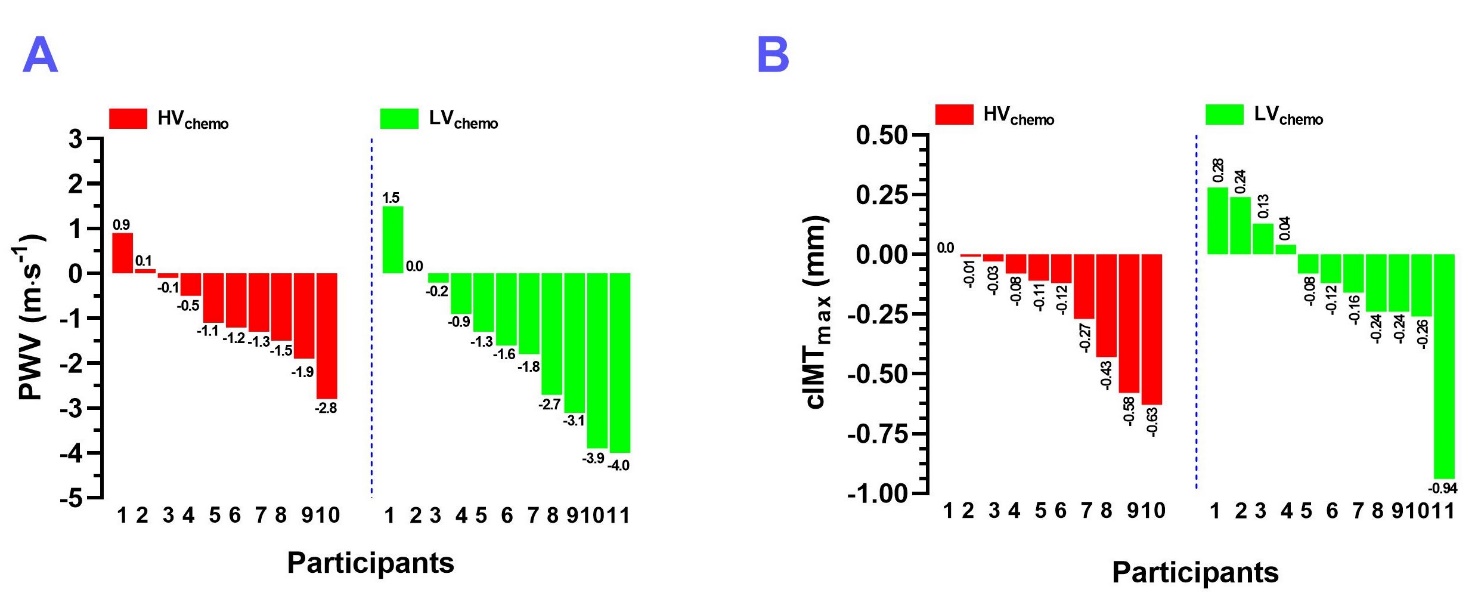


**Supplementary Figure S2.** Inter-individual variability after 8 weeks of concurrent training in breast cancer survivors in functional and structural vascular outcomes. **Groups are described as;** (Panel A and D; [HV_chemo_] High-volume chemotherapy group, (LV_chemo_) Low-volume chemotherapy group. **Outcomes are described as;** (PWV) Pulse wave velocity, and (cIMT_max_) Carotid intima-media thickness *maximum*.

**Supplementary Figure S3**


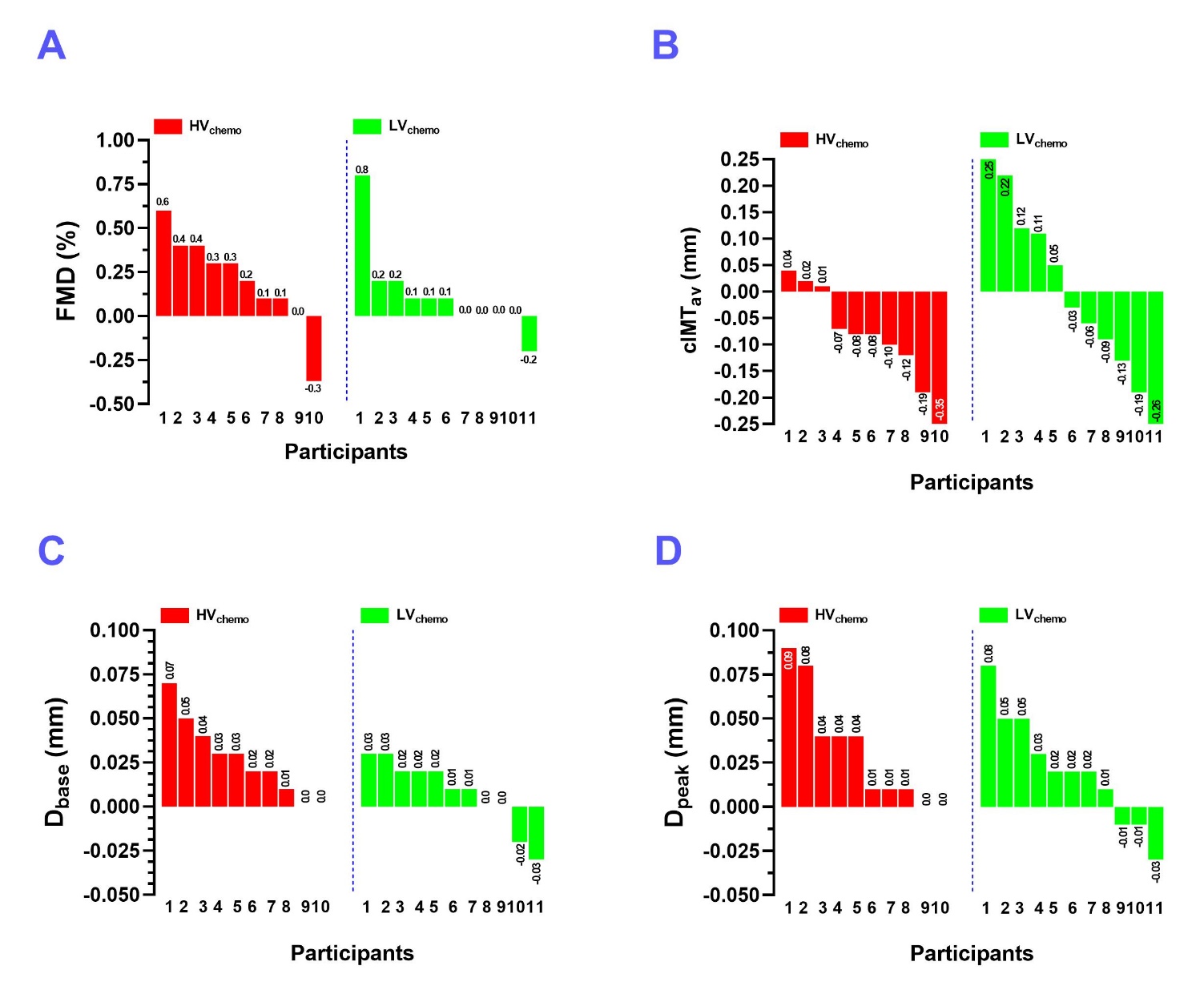


**Supplementary Figure S3.** Inter-individual variability after 8 weeks of concurrent training in breast cancer survivors in functional and structural vascular outcomes. **Groups are described as;** (Panel A and D; [HV_chemo_] High-volume chemotherapy group, (LV_chemo_) Low-volume chemotherapy group. **Outcomes are described as;** (FMD) Flow-mediated dilation, (cIMT_av_) Carotid intima-media thickness average, (D_base_) Brachial artery diameter at baseline, and (D_peak_) Peak brachial artery diameter after 5 minutes occlusion.

**Supplementary Figure S4**


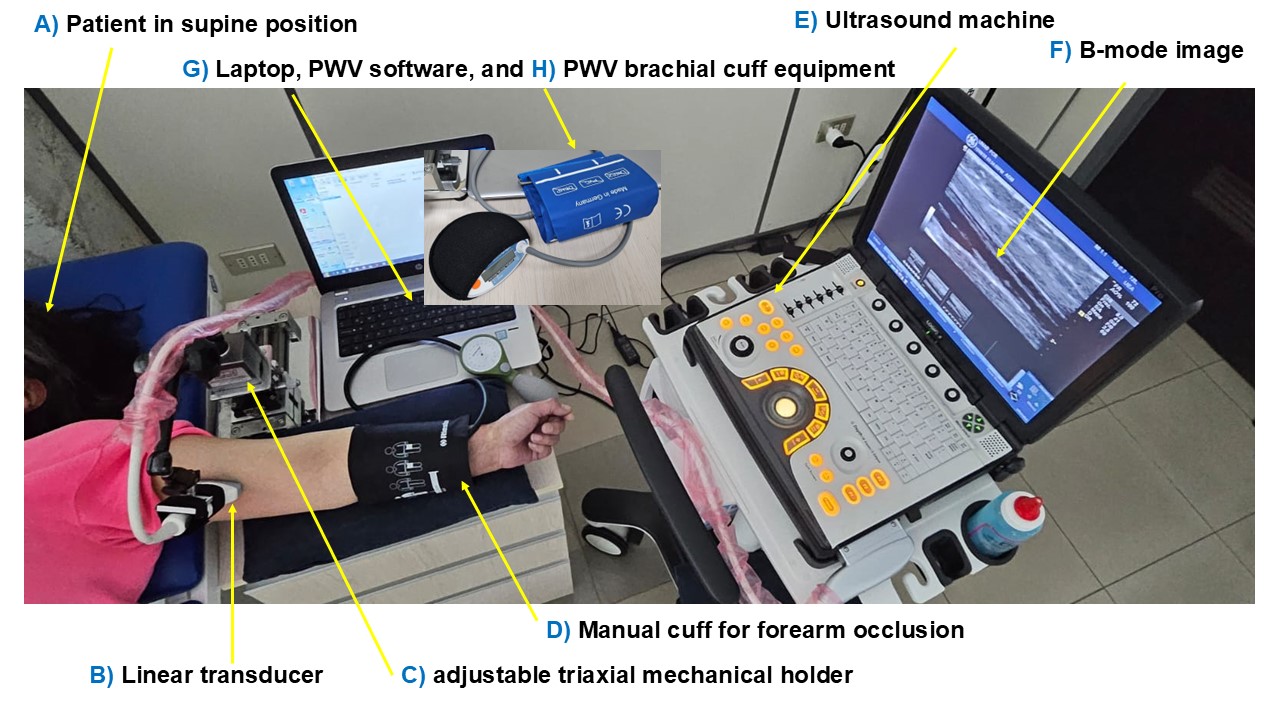


**Supplementary Figure S4.** Schematic image of flow-mediated dilation and pulse wave velocity measurement from the brachial artery.
